# Aphid-encoded variability in susceptibility to a parasitoid

**DOI:** 10.1186/1471-2148-14-127

**Published:** 2014-06-10

**Authors:** Adam J Martinez, Shannon G Ritter, Matthew R Doremus, Jacob A Russell, Kerry M Oliver

**Affiliations:** 1 Department of Entomology, University of Georgia, Athens GA 30602, USA; 2 Department of Biology, Drexel University, Philadelphia PA 19104, USA

**Keywords:** Resistance, Trade-off, Symbiont, Selection, Fecundity, Immunity

## Abstract

**Background:**

Many animals exhibit variation in resistance to specific natural enemies. Such variation may be encoded in their genomes or derived from infection with protective symbionts. The pea aphid, *Acyrthosiphon pisum,* for example, exhibits tremendous variation in susceptibility to a common natural enemy, the parasitic wasp *Aphidius ervi*. Pea aphids are often infected with the heritable bacterial symbiont, *Hamiltonella defensa*, which confers partial to complete resistance against this parasitoid depending on bacterial strain and associated bacteriophages. That previous studies found that pea aphids without *H. defensa* (or other symbionts) were generally susceptible to parasitism, together with observations of a limited encapsulation response, suggested that pea aphids largely rely on infection with *H. defensa* for protection against parasitoids. However, the limited number of uninfected clones previously examined, and our recent report of two symbiont-free resistant clones, led us to explicitly examine aphid-encoded variability in resistance to parasitoids.

**Results:**

After rigorous screening for known and unknown symbionts, and microsatellite genotyping to confirm clonal identity, we conducted parasitism assays using fifteen clonal pea aphid lines. We recovered significant variability in aphid-encoded resistance, with variation levels comparable to that contributed by *H. defensa.* Because resistance can be costly, we also measured aphid longevity and cumulative fecundity of the most and least resistant aphid lines under permissive conditions, but found no trade-offs between higher resistance and these fitness parameters.

**Conclusions:**

These results indicate that pea aphid resistance to *A. ervi* is more complex than previously appreciated, and that aphids employ multiple tactics to aid in their defense. While we did not detect a tradeoff, these may become apparent under stressful conditions or when resistant and susceptible aphids are in direct competition. Understanding sources and amounts of variation in resistance to natural enemies is necessary to understand the ecological and evolutionary dynamics of antagonistic interactions, such as the potential for coevolution, but also for the successful management of pest populations through biological control.

## Background

Insects and other animals face attack from a wide range of natural enemies which place strong selective pressures on the development, acquisition, and maintenance of resistance [1-10]. Intraspecific variation in resistance to natural enemies has been documented in many organisms and can stem from factors encoded in the host’s genome or those acquired from microbial associates [11-16]. Such variation is important for adaptation via natural selection, can promote the evolution of virulence in natural enemies, and drive host-enemy coevolutionary dynamics [13,17]. Resistance, however, may also be expected to carry costs as resources allocated to the defense are unavailable for other functions [18], such that resistance may result in decreased fitness under enemy-free conditions [19-24]. Furthermore, selection for host resistance can reduce enemy prevalence or encourage increased virulence, which in turn, can result in negative frequency-dependent selection against the now potentially costly, resistant phenotypes [11,25,26]. Variability in resistance may also be maintained by fluctuations in enemy pressure, variation in enemy virulence, host-enemy specificity, and mediating environmental factors [12,27-31]. Thus, quantifying variation and costs, and properly attributing the sources of variation in resistance to natural enemies, are often required to understand the ecological and evolutionary dynamics associated with antagonistic interactions, including those of economic concern, such as the successful deployment of biological control programs targeting pest organisms.

Insect-parasitoid interactions are among the most common antagonistic interactions in nature, where the survival of one player ultimately leads to the death of the other, resulting in strong selection for host resistance and parasite counter-resistance [32,33]. Interactions between the phloem-feeding aphids (Hemiptera) and their hymenopteran parasitoids have received considerable attention (rev. [34]). Many aphids, including the pea aphid, *Acyrthosiphon pisum,* reproduce parthenogenetically for most of the year, such that variation in resistance among clonal lines to particular natural enemies can be examined. An important study by Henter and Via [35] found that some North American pea aphid clonal lines were totally resistant to attack by the prevalent parasitoid *Aphidius ervi*, while other clones were highly susceptible. Subsequent work found that European pea aphid clones also varied in resistance to the wasp *A. ervi*, as well as the more host-specific congener *A. eadyi*[36-38]. It was first assumed that variation in resistance resulted from immunological pathways encoded in the aphid genome, but later studies found that infection with heritable bacterial symbionts was responsible for a substantial portion of the observed variation [39]. A number of studies identified correlations between infection with *Hamiltonella defensa* and increased clonal resistance to parasitism in the laboratory, but did not explicitly disentangle host- and symbiont-based effects (e.g. [40-42]). Simultaneous experimental studies, comparing aphid clones with and without symbionts, found that most *H. defensa* strains, and a single *Serratia symbiotica* strain, provided defense against the wasp *A. ervi*[39,43-45]. Further investigation of this interaction found that bacteriophages called APSEs were required for *H. defensa* to confer protection to pea aphids [43,46,47] and that levels of resistance to the wasp varied greatly and correlated with symbiont strain and associated virus type; uninfected aphid clones (i.e. no facultative symbionts), on the other hand, exhibited limited variation in resistance and were highly susceptible to attack [43,44]. Together, this work suggested that pea aphids primarily rely on infection with *H. defensa* and APSE to thwart attack from this common natural enemy. This hypothesis was bolstered by the observation that pea aphids have a weak encapsulation response to parasitism [48,49]. A recent study, however, found two pea aphid clones exhibited substantial resistance to *A. ervi* in the absence of *H. defensa* or other facultative symbionts [12] indicating that aphid-based resistance persists in North American *A. pisum* populations and may contribute more to the observed variation in susceptibility than is currently appreciated. Aphid-encoded resistance to parasitism has also been reported in the peach-potato aphid, *Myzus persicae,* and the black bean aphid, *Aphis fabae*[50-52].

To examine the extent of pea aphid encoded variability in resistance to parasitism by the wasp *A. ervi*, we conducted parasitism assays across a range of aphid clones that were devoid of facultative symbionts. We also estimated the fecundity and longevity of several clonal lines of varying resistance to determine whether increases in resistance correlated with reductions in fitness, which would be expected if resistance is energetically costly.

## Methods

### Study organisms

The pea aphid, *Acyrthosiphon pisum,* has diversified into numerous genetically distinct host races that specialize (i.e. have increased preference for, and performance on) on a variety of cultivated herbaceous legumes, including economically important crops such as alfalfa and clover [53-60]. This aphid was introduced to North America from Europe in the late 1800s [61], but native and introduced populations exhibit similar patterns of linkage disequilibrium, nucleotide diversity and symbiont diversity; together suggesting bottleneck effects have not limited diversity relative to source populations [62-64]. At most N. American latitudes this aphid is cyclically parthenogenetic and reproduction is asexual and viviparous for the majority of the year, with sexual morphs arising in the fall in response to shorter day lengths [65]. Clonal lines were maintained in the laboratory by rearing them under long day conditions. Each clonal aphid line used in this study (Table 1) was initiated from a single parthenogenetic female placed onto a caged broad bean plant, *Vicia faba,* and reared at 20 ± 1˚C with a 16 L: 8D photoperiod. We verified that all experimental aphid lines used in this study were free of facultative symbionts by using 1) diagnostic PCR to screen for all known pea aphid facultative symbionts, 2) ‘mostly universal’ PCR primers that amplify most bacteria, but not the obligate symbiont *Buchnera,* and 3) Denaturing Gradient Gel Electrophoresis (DGGE) with universal 16S rRNA bacterial primers. Primers, PCR cocktails and reaction conditions, and detailed DGGE protocols can be found in [64]; all PCR reactions contained positive and negative controls.

**Table 1:** Genetically distinct aphid clonal lines used in this study

**Aphid clone**	**Collection locale**	**Host plant**	**Reference**
5A	Wisconsin, USA 1999	Alfalfa	Sandstrom et. al. [66]
AS3-AB	Utah, USA 2007	Alfalfa	Martinez et. al. [12]
CJ1-13	Utah, USA 2012	Alfalfa	This paper
CJ1-15	Utah, USA 2012	Alfalfa	This paper
CJ2-6	Utah, USA 2012	Alfalfa	This paper
CJ4-2	Utah, USA 2012	Alfalfa	This paper
LSR01	New York, USA 1998	Alfalfa	Richards et. al*.*[67]
PB17	Pennsylvania, USA 2011	Alfalfa	This paper
WA4-AB	Pennsylvania, USA 2010	Alfalfa	Martinez et. al. [12]
WI27	Wisconsin, USA 2011	Alfalfa	This paper
WI48	Wisconson, USA 2011	Alfalfa	This paper
ZA17-AB	Pennsylvania, USA 2010	Alfalfa	Martinez et. al. [12]
BP14	Georgia, USA 2010	Crimson Clover	Parker et. al. [68]
G15	Georgia, USA 2008	Mixed Weeds	Parker et. al. [68]
G6	Georgia, USA 2008	Mixed Weeds	Barribeau et. al. [69]

The solitary endoparasitoid, *Aphidius ervi* (Hymenoptera: Braconidae), also introduced from Europe, is the most prevalent parasitic wasp attacking *A. pisum* populations in North America [70]. The wasps used in this study were obtained from a single, large, laboratory colony containing a mixture of *A. ervi* collected from Wisconsin and North Dakota, as well as commercially produced mummies (Arbico Organics). Wasps were reared continuously on a susceptible aphid line, AS3-AB; adults were provided with constant access to honey and water.

### Microsatellite analyses to distinguish clonal lines

Microsatellite genotyping was used to confirm the identity and genetic variability between clonal aphid lines used in this study. DNA extractions of each aphid line were performed using an Omega EZNA® Tissue DNA Kit and were stored at -20°C until use. Four microsatellite loci— Ap-02, Ap-03, Ap-05 [71], and Aph10M [72]—were PCR amplified with Dye Set-30 (DS-30) fluorescent primers using a touchdown reaction as follows: 94°C for 3 min; 45 cycles of 95°C for 30 s, 68–56°C touchdown for 13 cycles, then 55°C for 32 cycles, each cycle for 30 s, 72°C for 30 s; 72°C for final elongation, then held at 4°C. Fluorescent genotyping was then conducted by The Georgia Genomics Facility on an Applied Biosystems 3730xl DNA Analyzer, using the ROX500 size standard. Genotypic data were then analyzed using Geneious® version 6.1 (Biomatters).

Analysis of aphids typed at the four microsatellite loci revealed that all fifteen pea aphid lines used in this study represented distinct genotypes (See Additional file 1: Table S1 for details on loci used and allele sizes).

### Aphid parasitism resistance assays to determine protective phenotype

Parasitism assays to determine the resistance phenotype were carried out on all aphid lines used in this study (Table 1) as in [43]. Twenty 2nd to 3rd instar aphids were singly parasitized (each aphid is removed as it is parasitized) for each replicate (at least eight replicates) and placed on a fresh *V. faba* plant in a cup cage and held at 20 ± 1˚C and 50% relative humidity with a 16 L: 8D photoperiod. Prior studies have shown that isofemale lines of *A. ervi* wasps can vary in their counter-resistance (i.e. virulence), defined as their ability to successfully parasitize pea aphids [73], suggesting also that virulence, at least toward symbiont-mediated resistance, may evolve rapidly [74]. Although we have not observed substantial variation in wasp virulence (Oliver, personal observation), we designed our parasitism assays to minimize such potential effects. In short, utilized wasps were collected haphazardly from our large laboratory culture (see above), which was maintained on a highly susceptible clone, and numerous female wasps were used to singly parasitize each line. After nine days, we counted the number of live aphids, dead aphids, and aphid mummies (dried aphids containing a wasp pupa) to determine the proportion of each measured as: survival (live aphids/20), mortality (dead aphids/20), and mummification (aphid mummies/20). A large majority of adult wasps eclose from mummies making them a suitable proxy for determining levels of successful parasitism [75]. To determine background rates of mortality for each line, we placed five replicates of twenty *unparasitized* 2^nd^ to 3^rd^ instar aphids from each line on fresh plants and mortality was recorded from the control lines after nine days.

### Aphid fitness assays

We conducted cumulative fecundity assays under permissive conditions (no aphid or plant stresses) to investigate potential tradeoffs between parasitoid resistance and aphid fecundity. Six aphid lines, those with the most (WA4-AB, ZA17-AB, CJ1-13) and least resistance (G15, AS3-AB, LSR01) to *A. ervi* (See parasitism assay results), were selected for this assay. Prior to the experiment, each aphid line was reared on multiple plants, chosen haphazardly, from a cohort of healthy plants of similar age and size for several generations in 16 L:8D intervals at 20°C in a Percival I-41LLVL environmental incubator to reduce effects resulting from variation in prior culturing (i.e. maternal and grandmaternal effects). From these cultures, approximately twenty adults from each line were placed on a fresh plant and allowed to reproduce for 17 ± 1 h before removal. The resulting offspring were left to mature until they were 48 ± 8.5 h-old and then six nymphs (x8 replicates = 48 aphids per line) were placed in a cup cage containing a single *Vicia faba* plant. Cages were examined at 3-day intervals. At these times, the numbers of live and dead aphids of the original cohort were recorded to measure longevity, while the numbers of offspring produced were recorded to measure fecundity. Offspring were discarded at the time of counting to prevent their growth to maturity and subsequent offspring production as in [45].

### Statistical analyses

Aphid survival, mortality, and mummification (see above) were determined for each replicate of each parasitized aphid line. These values were used to compare differences among aphid lines using a Generalized Linear Model (GzLM), with a binomial distribution and logit link function. Survival, mortality, and mummification data were mildly overdispersed and so final test values are reported with a quasibinomial adjustment. A Post hoc Tukey’s HSD test on aphid survival, mortality, and mummification was performed using an ANOVA of arcsine transformed proportional data for pairwise comparisons among aphid lines. GzLM was also used to compare mortality of parasitized and control (unparasitized) aphids, both within and across lines (see Additional file 2: Figure S1). As aphid mortality after parasitism may be tied to differences among aphid lines, linear regression was performed on mean mortality between unparasitized and parasitized aphids among all lines used (see Additional file 2: Figure S1). Mean mortality was natural log transformed to satisfy normality assumptions of the linear regression.

Several analyses were employed to compare fitness parameters (fecundity and longevity) between aphids with high and low resistance to parasitism. Linear mixed models with heterogeneous first order auto regressive (ARH1) covariance structure (to account for repeated measures) were used to examine the effect of aphid line and resistance on cumulative aphid fecundity through several time points. Multivariate analysis of variance (MANOVA) with repeated measures design was used to examine the effect of aphid line and resistance on aphid longevity. All analyses comparing the effect of resistance on aphid fecundity or longevity were done by nesting ‘aphid genotype’ (six aphid lines) within ‘resistance’ (high or low).

## Results

### Parasitism assays

**Figure 1:**
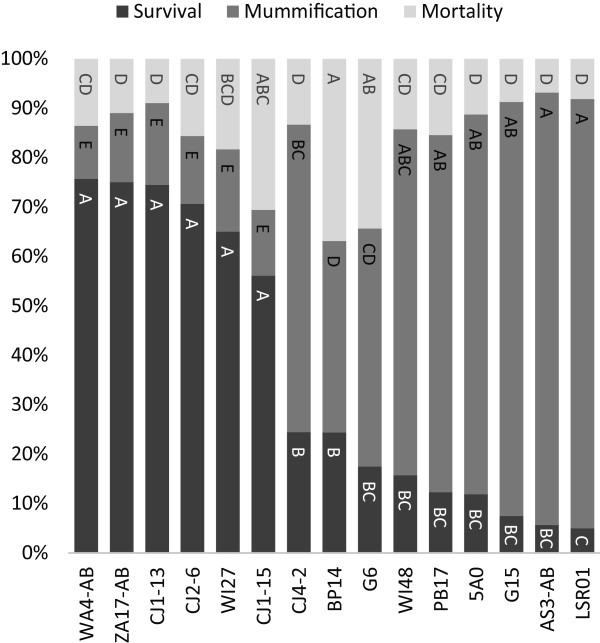
**Survival, mummification, and mortality rates of *A. pisum *counted nine days after parasitism by *A. ervi.*** Significant differences are indicated by letters (Tukey’s HSD α = 0.05). GzLM, df = 14, p < 0.0001 for all comparisons.

Parasitism by *A. ervi* results in three possible outcomes: wasps may complete development through pupation (i.e. aphid dies and is converted into a wasp “mummy”), aphids may survive parasitism and grow to adulthood, or both aphid and wasp may perish following parasitism. Among all fifteen pea aphid lines that were uninfected with facultative symbionts, we found significant variability in all three outcomes. From the aphid perspective, we find significant variation in their susceptibility to this important natural enemy (Survival: GzLM, χ^2^ = 488.2, df = 14, p < 0.0001) with survival rates ranging from 5 – 76%. Mortality (to both aphid and wasp) also varied significantly among lines (Mortality: 7 – 37%; GzLM, χ^2^ = 100.8, df = 14, p < 0.0001); however, in general, aphids that were not successfully parasitized (i.e. mummified) survived to adulthood (Figure 1). Mummification (successful parasitism) also varied among lines (Mummification: 11 – 88%; GzLM, χ^2^ = 424.6, df = 14, p < 0.0001) (Figure 1).

The majority of our *A. pisum* clones (12/15) were collected from alfalfa. To determine if there is significant variation in susceptibility among clones of this host race we conducted a restricted analysis and found similar variation in survival, mortality, and mummification (GzLM df = 11; χ^2^ = 389.2, 38.24, 344.82; p < 0.0001, < 0.0001, and < 0.0001; respectively).

Mortality also varied significantly among control lines not exposed to wasps (2 – 15%; GzLM, df = 14, χ^2^ = 24.45, p = 0.0404), and parasitism often resulted in significant increases in mortality relative to controls of the same line (Additional file 2: Figure S1). A linear regression analysis found no correlation between mortality of unparasitized controls and parasitized treatments (Linear Regression, F_1, 13_ = 2.02, p = 0.1784) indicating that parasitism-induced mortality affects clonal lines differently than their background mortality.

### Aphid fitness assays

**Figure 2:**
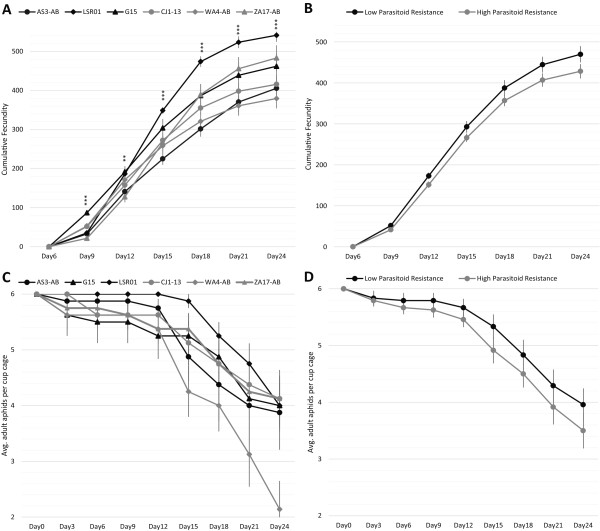
**Aphid fecundity and longevity among lines showing low (black) and high (grey) resistance to parasitism. A)** Cumulative fecundity for each aphid line. **B)** Cumulative fecundity between high and low parasitoid resistance groups. **C)** Longevity of each aphid line. **D)** Longevity between high and low parasitoid resistance groups. See Table 2 for tests and significance values. **p < 0.01, ***p < 0.001.

Total aphid fecundity, per replicate cup cage, measured over a twenty-four-day period revealed significant variation among the six (three high and low resistance) aphid lines tested (Figure 2A; Table 2A), but we found no inverse correlation (i.e. tradeoff) between resistance and fecundity (Figure 2B; Table 2B). We also estimated daily fecundity per live adult aphid (Additional file 3: Figure S2) and, again found significant variation among lines, but we did not find a significant association with the resistance phenotype. We also measured longevity of the original cohort of aphids for each line (Figure 2C; Table 2C) and between high and low resistance phenotypes (Figure 2D; Table 2D), but found no significant differences in either. Overall, in the absence of parasitism, resistant and susceptible lines exhibited similar fitness profiles, with no significant impact on longevity or fecundity owed to resistance phenotype.

**Table 2:** Effect of aphid genotype and resistance phenotype on fecundity and longevity

	**Aphid fecundity (Cumulative)**						**Aphid longevity**					
	**(A) Aphid line**			**(B) Resistance**			**(C) Aphid line**			**(D) Resistance**		
	**Df**	**F-Value**	**P-Value**	**Df**	**F-Value**	**P-Value**	**Df**	**F-Value**	**P-Value**	**Df**	**F-Value**	**P-Value**
Day 3	-	-	-	-	-	-	5,42	0.71	0.6220	1,42	0.06	0.8047
Day 6	-	-	-	-	-	-	5,42	0.71	0.6226	1,42	0.49	0.4885
Day 9	5,39.1	10.38	< 0.0001*	1,4.27	0.11	0.7593	5,42	0.64	0.6680	1,42	0.76	0.3893
Day 12	5,40.5	3.70	0.0075*	1,5.75	0.43	0.5384	5,42	1.30	0.2830	1,42	1.06	0.3090
Day 15	5,41.9	5.68	0.0004*	1,8.82	0.57	0.4691	5,42	2.17	0.0758	1,42	1.92	0.1728
Day 18	5,44.4	8.60	< 0.0001*	1,11.9	0.69	0.4214	5,42	0.95	0.4562	1,42	0.86	0.3595
Day 21	5,47.5	7.43	< 0.0001*	1,11.9	1.03	0.3310	5,42	1.15	0.3498	1,42	0.82	0.3709
Day 24	5,49.7	6.21	0.0002*	1,12.2	1.25	0.2859	5,42	2.24	0.0680	1,42	1.32	0.2568

Analysis of (A) the effect of aphid line on cumulative fecundity (linear mixed model), (B) the effect of resistance to parasitism on cumulative fecundity (linear mixed model), (C) the effect of aphid line on aphid longevity (repeated measures MANOVA), (D) the effect of aphid resistance on longevity (repeated measures MANOVA). Six aphid genotypes were analyzed, three susceptible and three resistant to parasitism. Note: Aphid offspring were not present until day nine. Significant values are indicated with an asterisk. See Figure 2 for graphs of analyses.

## Discussion

### Substantial variability in aphid-based resistance to parasitism

Pea aphids have been previously shown to exhibit substantial clonal variation in susceptibility to the parasitic wasp *A. ervi* (e.g. [35,36], with more recent studies showing that infection with the heritable protective symbiont *H. defensa* contributes much of the observed variation [12,43,44]. In these latter studies, a total of eleven uninfected clones were all highly susceptible to attack, while infection with *H. defensa* resulted in varying levels of protection correlating with symbiont strain and associated bacteriophage haplotype [12,43,44,75]. Due to the limited number of aphid clones used in these studies, however, it remained unclear whether there was also appreciable variation in resistance encoded by the aphid genotype. Using fifteen clonal pea aphid lines free of *H. defensa* and other facultative symbionts, we report here that there is indeed extensive aphid-encoded variation for resistance to *A. ervi* (Figure 1). In fact, the six most highly resistant clones (Figure 1) exhibit levels of defense (~55 – 75% survival) comparable to those contributed by defensive symbionts (~35 – 100% survival) [12,43,44,71].

These findings indicate that pea aphids employ both aphid- and symbiont-based strategies to aid in their interactions with this prevalent natural enemy. In addition to aphid- and *H. defensa* encoded protection, other common aphid symbionts, including *S. symbiotica*[39], or combinations of symbionts [42,45,76] show promise in influencing interactions with wasps. To date, however, the majority of uninfected clones (including those in this study: 21/27) examined in laboratory studies were found to be >65% susceptible to attack by this wasp [12,39,43,44,75] suggesting that symbiont-based protection may be the most frequently used line of defense. However, infection frequencies of *H. defensa* are quite variable (10 to 58%) in N. American field populations [64], and there may be dynamic temporal and spatial variation in the relative proportions of each mode of defense. This, of course, depends on the efficacy of symbiont- and aphid-based defenses under natural conditions, which is largely unknown [77,78]. Temperature, for example, is known to affect *A. pisum* clonal resistance to parasitism and appears to be due primarily to losses in *H. defensa*-mediated protection at higher temperatures [40], but these assays were not conducted while controlling for aphid genotype and hence it is possible that temperature also impacts aphid-encoded resistance. If higher temperature indeed impacts symbiont-based resistance to a greater degree, then we would expect fewer *H. defensa*-bearing aphids and more aphid-encoded resistance in warmer regions and seasons.

The majority of clones we examined were collected from alfalfa (Table 1), and an analysis restricted to this host race also recovered substantial variation in susceptibility to parasitism (Figure 1). Additional studies are required to determine if there is substantial aphid-encoded variation in susceptibility *within* populations or whether there is geographical variation among collection sites. We did not detect significant variation among sites in this study, but the sampling was very limited. Facultative symbiont infection frequencies are known to vary among pea aphid host races, and infection with *H. defensa* occurs more frequently on alfalfa than on other host plants [63,64]. We might predict that aphid-encoded defenses against *A. ervi* are more common in other host races, such as clover, with lower *H. defensa* infection frequencies. One study, however, reported that variation in aphid resistance (due to any mechanism) was much lower on clover (~60 - 95% susceptible), compared to alfalfa (~5 – 90% susceptible), and that clover clones were generally more susceptible than alfalfa clones to parasitism by *A. ervi*[59]. One possible explanation for the presence of both higher *H. defensa* infection frequencies and more aphid-encoded protection on alfalfa is that clover-derived *A. pisum* suffer reduced rates of attack under field conditions resulting in less selection pressure for the evolution and maintenance of resistance. However, we caution that further work is needed to evaluate the ranges of aphid-encoded resistance on clover (and other host races) and the importance of the various resistance components under field conditions.

The mechanisms underlying this aphid-based resistance to parasitism are unknown. The pea aphid lacks a strong encapsulation response, the innate cellular immune response used by many insects to encapsulate and asphyxiate invading parasitoid eggs [48,49,79]. Sequencing of the pea aphid genome revealed several pathways (e.g. IMD) involved in innate immunity against pathogenic microbes were missing, yet this aphid retains important pathways associated with encapsulation [80] and are able to melanize foreign objects [49]. We are currently investigating the phenology and mechanisms underlying both symbiont- and aphid-based immunity to this wasp.

Pea aphids also show clonal variation in susceptibility to the aphid-specific fungal entomopathogen *Pandora neoaphidis* (e.g. [36]). The heritable symbiont *Regiella insecticola* and other symbionts have been shown to confer protection against this and other specific fungal pathogens and thus contribute to variation in resistance [81-83]. More recent work indicates that pea aphids also show aphid-based clonal variation in their susceptibility to *Pandora*[68], indicating both aphid- and symbiont-based defensive strategies are utilized against diverse natural enemies. It will be interesting to determine if there is a negative correlation in resistance to parasitoids and fungal pathogens, providing a potential explanation for the persistence of susceptible genotypes. In addition, we are seeking to determine whether resistant genotypes are less likely to carry protective symbionts, as services are duplicated. Recently, two *H. defensa* strains were found not to confer any additional protection beyond that of their resistant host aphid genotype [12] suggesting this may be the case, although it is also possible these strains would confer protection in a susceptible background, but that benefits are not additive. Duplication in defense, though, could partially explain why the beneficial symbiont *H. defensa* is not more prevalent in field populations [3,12,78].

The black bean aphid, *Aphis fabae*, also shows variation in resistance to its common parasitoid *Lysiphlebus fabarum*, with some variation encoded by the defensive symbiont *H. defensa*[84] and some likely encoded by the host genome [52]. Other aphids, including *Aphis craccivora* and *Myzus persicae* show clonal variation in susceptibility to parasitoids, including evidence for both symbiont- and host-encoded resistance [50,51,85] indicating that aphids generally use a variety of mechanisms to aid in their defense.

Populations of the parasitoid *A. ervi* have also been shown to exhibit variation in the ability to successfully parasitize pea aphids [73], but further work in this system is needed to determine whether there is variation in counter-resistance toward particular components of aphid defense. Such specificity in genotype by genotype interactions may be directed toward either aphid- or symbiont-based components of resistance, and while duplicated services may not provide an advantage against the average wasp genotype, it may provide protection against a wider range of enemy genotypes. As mentioned above, the wasp *A. ervi* appears capable of evolving virulence toward symbiont-based protection [74], but it is unclear if it can do so against aphid-based defenses. Such genotype by genotype interactions have been best studied in the black bean aphid-*H. defensa-L. fabarum* interaction, where they occur between parasitoid genotypes and defensive symbiont strains, but have not been found between parasitoid and uninfected host genotypes [52,86,87]. If wasps more readily evolve counter-resistance to symbiont-encoded resistance, this may lead to an increase in the frequency of *H. defensa*-free resistant clones in natural populations, or vice versa.

### No apparent trade-offs between parasitoid resistance and fitness

The maintenance of clonal variation in pea aphid susceptibility to the parasitoid *A. ervi* could be explained by tradeoffs in other functions given limited resources. Aphids, including *A. pisum*, have evolved a number of life history traits associated with increasing reproductive output, including cyclical parthenogenesis, wing polyphenisms, and telescoping generations [88]. Thus, if aphid-based resistance to parasitism carries constitutive costs, then we might expect to see a negative correlation between resistance and fecundity or longevity. While we did find significant clonal variation in fecundity, we did not find a positive correlation between susceptibility and fecundity or longevity among the most and least resistant lines (Figure 2; Table 2). Tradeoffs between resistance and aspects of host fitness, including development time, survival, and fecundity have been observed in other systems [20,22-24], but they are often difficult to detect in aphid systems [36,51]. One study [37] did find a tradeoff between resistance and fecundity among ten clonal pea aphid lines, but it is unclear if resistance was symbiont or aphid-based.

One possible reason we did not find the expected trade-off is that costs are induced rather than constitutive, such that costs are only manifested upon attack. We are currently investigating fecundity among parasitized clonal lines and other sub-lethal effects of parasitism, but preliminary trials indicate parasitized aphids that survive have similar fecundity to unparasitized controls (AJM unpublished). It is also possible that tradeoffs may only become apparent under more stressful conditions or when clones are in direct competition for resources, as our lab assays were conducted using lines held separately and reared under very permissive conditions [3]. For example, Kraaijeveld and Godfray [21] found trade-offs resulting from increased parasitoid resistance in *Drosophila melanogaster,* but these were only observable under high intraspecific competition for food resources. Costs associated with *H. defensa*-mediated resistance have also been difficult to detect in component fitness assays. Only when *H. defensa*-infected and uninfected lines sharing the same genotypes were reared together in population cages were costs to infection identified [3]. Thus, costs may become apparent under more realistic conditions, with varying biotic (e.g. plant quality) and abiotic factors (e.g. water stress, temperature), and when intra- and interspecific competition is present.

## Conclusions

Here we show that pea aphid genomes maintain variation in susceptibility to a common natural enemy, the parasitoid *A. ervi*. Together, with prior work showing that infection with the heritable symbiont *H. defensa* confers varying levels of protection, depending on strain and phage type, it is clear that this aphid employs multiple strategies to thwart attack from parasitoids. It remains unclear whether resistant aphid genotypes and protective symbionts like *H. defensa* interact, or whether effects are additive or redundant, as this would be an important factor influencing the spread of symbiont- and aphid-based resistance in natural populations. It is important to understand the sources and amount of variation in resistance to common natural enemies, and how each is impacted by biotic and abiotic interactions. Temperature, for example, may differentially influence wasp and aphid behavioral responses and also affect the performance of aphid- and symbiont-encoded resistance [75] depending on presence and type of defense. Multiple sources of resistance may limit the evolution of resistance (when, for e.g., both types are employed in same host) or generate complex genotype by genotype interactions where some wasp genotypes specialize on particular aphid clone-symbiont strain combinations. Understanding the sources and dynamics of resistance is also important for the effective management of pest populations. If resistance is due primarily to symbionts, for example, then a quick diagnostic screen may inform whether biological control applications are likely to be effective.

### Availability of supporting data

The data sets supporting the results of this article are available in the Dryad repository [89], http://dx.doi.org/10.5061/dryad.6b5f0.

## Competing interests

The authors declare that they have no competing interests.

## Authors’ contributions

AM and KO designed the experiments. AM, SR, and MD performed the experiments. AM performed the microsatellite analyses, all statistical analyses, and created all figures and tables. AM, KO, and JR wrote the manuscript. All authors read and approved the final manuscript.

## Supplementary Material

Additional file 1: Table S1 Allele sizes for four microsatellite loci. Click here for file

Additional file 2: Figure S1 Mortality rates (excluding mummification) between parasitized and control (unparasitized) aphid lines, nine days after parasitism. *p < 0.05, **p < 0.01, ***p < 0.0001. Click here for file

Additional file 3: Figure S2 Average fecundity per aphid per day, among lines showing high (solid bars) and low (striped bars) resistance to parasitism. Letters indicate significant differences within a single day (Tukey’s HSD α = 0.05). Linear mixed model, F_5,237_, p < 0.01 for each day. Click here for file
