# Supplementary material for: Aphid-encoded variability in susceptibility to a parasitoid
Source: BMC Evol Biol. 2014 Jun 10;14:127. doi: 10.1186/1471-2148-14-127 (PMC4057601; doi:10.1186/1471-2148-14-127)
Supplement: Additional file 1: Table S1 — Allele sizes for four microsatellite loci. [file 1471-2148-14-127-S1.doc]

**Supplemental Table 1**. **Allele sizes for four microsatellite loci.**

|  | **Ap02** | | **Ap03** | | **Ap05** | | **Aph10M** | |
| --- | --- | --- | --- | --- | --- | --- | --- | --- |
|  | **allele 1** | **allele 2** | **allele 1** | **allele 2** | **allele 1** | **allele 2** | **allele 1** | **allele 2** |
| 5A0 | 228 | 232 | 242 |  | 265 |  | 198 | 202 |
| AS3-AB | 228 |  | 242 |  | 265 | 271 | 198 | 202 |
| BP14 | 228 |  | 240 | 242 | 265 | 267 | 194 |  |
| CJ1-13 | 228 | 232 | 240 | 242 | 267 |  | 196 | 198 |
| CJ1-15 | 232 |  | 240 | 242 | 253 | 265 | 194 |  |
| CJ2-6 | 228 | 232 | 240 | 242 | 267 |  | 194 |  |
| CJ4-2 | 228 | 232 | 242 |  | 267 |  | 194 | 198 |
| G6 | 228 | 232 | 242 |  | 265 | 267 | 194 |  |
| G15 | 228 |  | 236 | 240 | 267 | 271 | 194 | 202 |
| LSR01 | 222 | 228 | 242 | 256 | 265 |  | 194 |  |
| PB17 | 232 |  | 242 |  |  |  | 194 | 198 |
| WA4-AB | 228 | 232 | 242 | 256 | 265 | 267 | 194 |  |
| WI27 | 232 |  | 240 | 242 | 265 | 267 | 194 | 198 |
| WI48 | 232 |  | 242 |  | 265 |  | 194 | 198 |
| ZA17-AB | 228 |  | 240 | 256 | 253 | 257 | 194 |  |

Blanks in the second column for each locus indicate homozygosity. Line PB17 did not amplify at the AP05 locus.
